# Postpartum Haemorrhage and Eclampsia: Differences in Knowledge and Care-seeking Behaviour in Two Districts of Bangladesh

**DOI:** 10.3329/jhpn.v27i2.3328

**Published:** 2009-04

**Authors:** Nahid Kalim, Iqbal Anwar, Jasmin Khan, Lauren S. Blum, Allisyn C. Moran, Roslin Botlero, Marge Koblinsky

**Affiliations:** ^1^ Public Health Sciences Division, ICDDR,B, GPO Box 128, Dhaka 1000, Bangladesh; ^2^ American Embassy Kinshasa, Unit 2220, Box 178, DPO, AE 09828-0178, USA; ^3^ Women's Health Program, Department of Medicine, Monash University, Melbourne, Australia; ^4^ John Snow Inc., 1616 Ft Myer Drive, Arlington, VA 22209, USA

**Keywords:** Beliefs, Care-seeking behaviour, Maternal mortality, Postpartum haemorrhage, Eclampsia, Qualitative research, Bangladesh

## Abstract

In high- and low-performing districts of Bangladesh, the study explored the demand-side of maternal healthcare by looking at differences in perceived knowledge and care-seeking behaviours of women in relation to postpartum haemorrhage or eclampsia. Haemorrhage and eclampsia are two major causes of maternal mortality in Bangladesh. The study was conducted during July 2006–December 2007. Both postpartum bleeding and eclampsia were recognized by women of different age-groups as severe and life-threatening obstetric complications. However, a gap existed between perception and actual care-seeking behaviours which could contribute to the high rate of maternal deaths associated with these conditions. There were differences in care-seeking practices among women in the two different areas of Bangladesh, which may reflect sociocultural differences, disparities in economic and educational opportunities, and a discrimination in the availability of care.

## INTRODUCTION

Some 12,000 women die every year from birth-related complications in Bangladesh (1). Three-quarters of these maternal deaths occur during labour, childbirth, or in the postpartum period (2). Postpartum haemorrhage (PPH) and eclampsia, accounting for 34% and 29% respectively, are major causes of these deaths (3). Global experience also suggests that the most common cause of maternal death is severe bleeding (25%) while another 12% of mothers die of eclampsia (4). These complications can be managed and treated if timely and appropriate care is sought from facilities with necessary skilled care providers.

Almost 85% of births in rural Bangladesh occur in the home with the vast majority delivered with the assistance of traditional birth attendants (TBAs) called *dais* (5,6). These unskilled care providers are not trained to handle life-threatening obstetric complications. The strong preference for home-birth is commonly associated with restrictions on female mobility and cultural norms (7-9). In the context of rural Bangladesh, behaviour during childbirth is largely influenced by an interplay of factors relating to the cultural constructions of pregnancy and childbirth, practical realities resulting from women's roles and status in society, and accessibility to healthcare services (2). In addition, referral rates for emergency obstetric care (EmOC) are typically low (10, 11). In the case of care-seeking in PPH, influential elderly women in the family were more interested to refer to the village doctor (pharmacy shop-keeper) or *kobiraz* (spiritual healer) than to a trained doctor (10).

Therefore, there is a tragic discontinuity among the onset of the problem, recognition of a dangerous condition, timing of the response, attendant's skill, and healthcare-seeking, all too often resulting in maternal death (10). Understanding and addressing how women and families recognize and respond to these potentially life-threatening complications is critical to devising improved safe motherhood programmes that are more accessible to women living in rural settings.

This paper examines how knowledge and care-seeking behaviours relating to obstetric haemorrhage and eclampsia differ in two areas of Bangladesh with contrasting levels of maternal mortality ratios (MMRs) and use of maternal healthcare services. We present the findings from qualitative research designed to: (a) understand the recognition of and response to PPH and eclampsia by women and (b) explore healthcare-seeking behaviours among those who experienced these complications. The overall aim of the study was to understand the varying responses in the study districts—Sylhet and Jessore−and identify information that could improve the response to these two major causes of maternal death.

## MATERIALS AND METHODS

### Study sites

Bangladesh comprises six divisions, covering 64 districts. Khulna and Sylhet divisions were selected as higher- and lower-performing divisions of the country in a national-level stakeholders' meeting convened in December 2004. Khulna division has an MMR of 351 and high-skilled attendance (20.5%) while Sylhet is an area with an MMR of 471 and low use of skilled attendance (10.1%) (3). In the higher-performing areas, the contraceptive prevalence rate (CPR) and female literacy were 63.8% and 68.0% while the female literacy rates for the lower-performing areas were 31.8% and 47.0% respectively. The rate of female education in Khulna is also two times higher than that of Sylhet (28.2% vs 14.9%) (3), which influences the degree of mobility of women, among other things. In general, rural women of Bangladesh are burdened with lower literacy rates, poor economic conditions, and lower social status than their urban counterparts (3,12,13). We selected four subdistricts from these two divisions—Abhaynagar and Keshabpur from Jessore and Zakiganj and Beanibazar from Sylhet—based on the availability of ICDDR,B data on women's age and parity. Women of reproductive age (15-49 years) and older (>50 years) and who had had a birth in their lifetime were included in the study.

In both the areas, care providers included those in public-sector comprehensive essential obstetric care (EOC) facilities and those private clinics, plus non-trained service providers, such as village doctors (pharmacy shop-keepers), herbalists, and religious healers. The study was undertaken over a 17-month period from July 2006 to December 2007.

### Study methods and sampling

We used a mix of qualitative research methods, including free-listing, rating exercises, hypothetical case scenarios, and in-depth interviews. These methods allowed us to explore the most commonly-perceived complications, their relative perceived severity, knowledge about the signs/symptoms of hypothetical cases of PPH and eclampsia, care-seeking behaviours, and also actual recognition of and response to PPH and eclampsia confirmed by skilled care providers and subsequent care-seeking behaviours.

Free-listing exercises were undertaken with both women of reproductive age (15-49 years) and elderly women (50-70 years) who had at least one birth experience in their lifetime. We selected these two age-groups to explore commonly-perceived complications and responses according to study area and age. Free-listings were carried out with 80 respondents chosen randomly in each site: half of the respondents were from the elderly group (n=20 in Sylhet and n=20 in Jessore) while the other half were women of reproductive age (n=20 in Sylhet and n=20 in Jessore). Addresses of the respondents were identified through the household listing and pregnancy surveillance system from Sylhet and from the sample registration system (SRS) in Abhaynagar, Jessore. Without prompting, the respondents were asked to list the most common complications or conditions associated with labour, childbirth, and the early postpartum period (within 48 hours after delivery). Each exercise took about 30 minutes.

Based on the findings of the free-listing, severity-rating exercises were then conducted in both the study areas, with the women of reproductive age who completed the free-listing (n=20 in each area). These exercises were designed to assess the perceived importance of PPH and eclampsia in relation to other complications during labour, childbirth, and the early postpartum period. All conditions were included in the ranking exercise the respondents were asked to rate each condition as severe, intermediate, or not severe in relationship to risk of death from that condition (with 1 being most severe and 3 being least severe). These interviews took about 80 minutes.

Hypothetical ‘case scenarios' were used for eliciting local knowledge and attitudes about signs associated with bleeding and eclampsia that triggered recognition and for identifying perspectives about their cause and treatment. Four scenarios—two involving a case of eclampsia and two involving postpartum haemorrhage—were developed. Every respondent was verbally presented with two of the four scenarios—one on PPH and another on eclampsia. Specific questions on signs/symptoms, causes, and first care-seeking actions were asked to women. Interviews for the hypothetical case scenarios took 80 minutes.

To assess real experiences relating to the recognition of and response to these two complications, 38 in-depth interviews were administered individually with those who had experienced PPH and/or eclampsia and their caregivers, e.g. mother-in-law, sister-in-law, mother, and TBA, who witnessed these events.

Those who experienced either of these complications during July 2006−December 2007 were selected purposively from a register kept in the government health centres of both the subdistricts (no community-level data were available). To understand the recognition and response to PPH and eclampsia, we selected women who experienced PPH and delivered in the home but later went to a health centre and those eclampsia patients whose convulsion started in the home but later received healthcare from the government health centres. In-depth interviews carried out with women who had experienced PPH and eclampsia were tape-recorded and later transcribed. Interviews were conducted in Bangla and translated into English. Each in-depth interview was conducted at a time and place convenient for the informant and took approximately 90 minutes.

The numbers of actual cases in both the sites were very few. For PPH, this study selected only one subdistrict of Jessore where the respondents did not have access to interventions offering misoprostol; from this one subdistrict, only four women with PPH were identified. Another limitation were that we knew little about the quality of care offered by different types of facilities and care providers.

Table 1 presents the sampling framework for each research method included in the study.

**Table 1.** Methods and total sample from two study sites, Sylhet and Jessore, 2006-2007

Method	Sylhet		Jessore	
	Women of reproductive age (15-49 years)	Elderly women (50-70 years)	Women of reproductive age (15-49 years)	Elderly women (50-70 years)	Total sample	Selection procedure
Free-listing	20	20	20	20	80	Randomly
Rating	20	-	20	-	40	Randomly
Hypothetical case scenario	20	-	20	-	40	Randomly
In-depth interview	Women who experienced	Care givers	Women who experienced	Care givers		
PPH	5	5	4	4	8
Eclampsia	5	5	5	5	20	Purposively

PPH=Postpartum haemorrhage

### Data collection

Five trained bilingual research officers with extensive experience in qualitative data collection administered all the interviews. Detailed notes were taken during the interviews and expanded shortly after the interview was completed.

After successful completion of each interview, a supervisor listened to the recorded versions and cross-checked transcriptions. The supervisor provided quick feedback on the quality of collected data to ensure quality data-collecting procedures.

### Analysis

Anthropac® 4 was used for analyzing the free-listing and rating data. In the free-listing exercises, conditions mentioned first and more often were considered to have greater saliency (14). Salience was a measure of how much knowledge informants share and how important that knowledge was to them. It was a score using the frequency and rank of each response from the free-listing data (15). Univariate analysis was conducted for the rating exercises. Hypothetical cases and in-depth interviews were coded using Atlas/Ti. Content analysis was done to identify patterns from the collected information.

The Ethical Review Committee of ICDDR,B approved the study protocol. Informed written consent was taken from all the respondents who participated in the study.

## RESULTS

### Free-listing: perceptions of the most salient conditions

The free-listing exercise produced 32 perceived complications relating to labour, delivery, and the immediate postpartum period. Table 2 and 3 list the 10 items with the highest salience reported by the respondents in both the study areas. Those that ranked 1 were the most salient.

**Table 2.** Rank order of perceived complications relating to labour, delivery, and the postpartum period reported by women of reproductive age and elderly female respondents, Sylhet and Jessore, 2006

Condition	Rank-order by age-group and site			
	Women of reproductive age (15- 49 years)		Elderly female respondents (50-70 years of age)	
	Sylhet (n= 20)	Jessore (n=20)	Sylhet (n=20)	Jessore (n=20)
Eclampsia	1	7	3	9
Prolonged labour	2	1	2	8
Prematurely ruptured membrane	3	-	-	6
After-pain	4	4	6	3
Postpartum haemorrhage	5	3	8	7
Antepartum haemorrhage	6	-	-	-
Retained placenta	7	5	5	2
Prolonged labour (24 hours)	8	-	-	-
Prolapsed hands	9	-	-	-
High fever	10	9	9	-
Tetanus	-	2	1	1
Anaemia	-	6	-	-
Baby is stuck	-	8	-	-
Fistula	-	10	-	-
Prolapsed uterus	-	-	-	5
Breech presentation	-	-	4	-
Transverse position	-	-	7	-
Weakness	-	-	-	4
No pain	-	-	10	-
Tear	-	-	-	10

**Table 3.** Severity rating of obstetric complications associated with risk of death by women of reproductive age, Sylhet and Jessore, 2006

	Sylhet			Jessore	
Illness	Average rating for each item	Ranking based on rating of severity	Illness	Average rating for each item	Ranking based on rating of severity
Retained placenta *(Aauda ashe na)*	1.05	1	Torn placenta *(Full chira jaowa)*	1.10	1
Tear placenta *(Chera aauda)*	1.10	2	Prolapsed hands *(Haat age asha)*	1.30	2
Postpartum haemorrhage *(Khun chuta)*	1.15	3	Tetanus *(Dhonustonkor)*	1.35	3
Antepartum haemorrhage *(Age khun chuta)*	1.15	3	Eclampsia *(Kichuni)*	1.45	4
Eclampsia*(Chikani beram*)	1.25	4	Fistula *(Paykhana-prosraber rasta ek hoya)*	1.45	4
Tetanus *(Dhonustonkor)*	1.30	5	Anaemia *(Rokto sunnota)*	1.50	5
Fistula *(Paykhana-prosraber rasta ek hoya)*	1.30	5	Antepartum haemorrhage *(Age rokto jaowa)*	1.55	6
Breech position *(Upta batcha)*	1.35	6	Postpartum haemorrhage *(Rokto aowa)*	1.60	7
Transverse position *(Pathailla batcha)*	1.40	7	EDD over *(Date par hoya gele)*	1.65	8
Premature labour *(Agam bedna)*	1.45	8	Retained placenta *(Full ashe na)*	1.65	8
Anaemia *(Khun nai)*	1.50	9	Prolonged labour *(Mela derir bedna)*	1.70	9
Tear *(Chira/Chera)*	1.55	10	Pain not strong enough *(Bedna beshi soktto na)*	1.85	10

Rating: 1=Most severe; 2=Intermediate; 3=Least severe; EDD=Expected date of delivery

Eclampsia was most frequently mentioned by women of reproductive age in Sylhet whereas, in Jessore, eclampsia was ranked only seventh out of 10. PPH occupied fifth position in Sylhet and third in Jessore. Among elderly women, eclampsia was in the third place in Sylhet and ninth in Jessore. PPH was listed in the eighth position in Sylhet and seventh in Jessore according to the frequency of mention.

### Severity rating: perceived severity of most salient conditions

In the risk-rating exercise, women of reproductive age from both the study sites rated 32 common complications on perceived severity; the complications were determined from the free-listing exercise.

Table 3 presents 10 complications viewed as most likely to lead to death during labour and delivery based on the severity-rating exercise. Eclampsia was perceived to be the fourth most dangerous complication in both the sites, and PPH was the third most severe obstetric risk in Sylhet and seventh in Jessore.

### Case scenarios: knowledge of danger signs and care-seeking for PPH

Stories and results of the hypothetical ‘case scenarios' on PPH are presented below.

Scenario A: Postpartum haemorrhage (four hours after delivery)

Fatema had already delivered three children. Things had gone well during this pregnancy. When she started to experience contractions, Fatema did not inform her family members. However, after a day, the labour pain became unbearable; so, she notified her mother-in-law. Fatema remained in labour for two days and finally, on the third day, delivered a baby boy. Immediately after delivery, her mother-in-law noticed that her clothes and bedding were soaked with blood. A lot of bleeding continued for four hours. Her eyes and face became pale. Fatema was very weak and had difficulty moving.

Scenario B: Postpartum haemorrhage (two days after delivery)

Aisha went into labour at 7:30 am. Her mother-in-law noticed that she was in pain and sent somebody to call the local *dai*, an older female relative who lived in the same bari. The *dai* arrived shortly afterwards, and she assisted Aisha to deliver a baby girl around the time of Zohar prayer at 12:45 (mid-day). After the delivery, Aisha experienced severe abdominal pain, and the placenta failed to come out. After half an hour passed, the *dai* tried to remove the placenta with her hand. She was able to remove only pieces of the placenta. She told the family members that the rest of the placenta would come gradually with the other discharge that women expel naturally after childbirth. After this procedure, Aisha was bleeding, and over a 24-hour period, the bleeding increased. The following day she became unconscious.

The interpretation of hypothetical case scenarios—both A and B—was that the first cause of heavy bleeding was reported to be ‘torn placenta' and ‘weakness from not having nutritious food'. Other causes of PPH were prolonged labour and malnutrition (Table 4). Only two of the 20 respondents in Sylhet mentioned that they would adopt further steps to take the patient to a hospital or call a doctor at home if they found ‘bleeding with half of the placenta not coming out', and 13 reported that they would take action when there is ‘heavy bleeding' (Table 4). Seven respondents in Jessore reported that they would take further steps in the case of ‘bleeding with half of the placenta not coming out', and another seven reported that they would do so if the women started to turn pale.

**Table 4.** Perceptions of causes and signs/symptoms of bleeding by women of reproductive age based on hypothetical case scenarios, Sylhet and Jessore, 2006

Cause, sign/symptom	Sylhet (n=20)	Jessore (n=20)
Physical		
Torn placenta	8	6
Prolonged labour	3	--
Weakness	1	7
Malnutrition	-	3
Do not know	6	1
Evil spirit	2	2
Allah's will	--	1
First-line treatment		
Home	1	7
Facility	1	3
Sign-prompting action		
Heavy/huge bleeding	13	2
Bleeding with half of the placenta do not come out	2	7
Bleeding with pale face/body	3	7
Bleeding with unconscious/faint	2	4
Bleeding with weakness and cannot move	3	--

∗Multiple responses were permitted

In both the sites, the respondents reported not only physical but also non-physical causes of PPH. ‘Evil spirits' were mentioned in both the areas while fate (Allah's will) was mentioned as a causal explanation in Jessore.

One respondent in Sylhet said:

*Dushi* (evil spirit) turns on a pregnant woman if she stays outdoors in the early morning, in the evening, and at noon, ignoring rules, and heavy bleeding occurs in this case.

Providing care at home was the first consideration in the case of bleeding. In the case of PPH, service providers called were qualified (MBBS) doctors, untrained village doctors, religious healers, and herbalists. The only difference between the respondents of the two sites was in the time needed to transfer the patient to a facility. In Sylhet, the women reported that they would call a trained doctor to their home within one hour whereas the respondents in Jessore reported that they would prefer taking the patient to a hospital within half an hour of the time the condition was recognized (Table 5).

**Table 5.** Perceptions of care-seeking associated with bleeding by women of reproductive age based on hypothetical case scenarios, Sylhet and Jessore, 2006

Care-seeking	Sylhet (n=20)	Jessore (n=20)
How quick should action be taken		
Within one hour	11	4
Within half an hour to one hour	5	5
Within half an hour	3	10
As early as possible	1	1
What type of practitioner chosen and why		
Home		
Call an MBBS doctor	7	6
Provides appropriate medicine		
Can refer patient on time		
Call village doctor (not medically trained)		
Available and experienced	3	4
Starts treatment early as he/she stays closer		
Bring *‘pani pora'* (blessed water) from *huzur* (Muslim spiritual healer who give spiritual blessings)	2	1
Blessed water helped to cure from evil spirit		
Call *kobiraj* (traditional healer, usually providing herbal treatment)	-	2
Available, provides good treatment to cure from evil spirit		
Facility	6	5
Go to hospital		
Getting free treatment		
Getting free medicine		
Getting specialized doctor		
Go to clinic	2	2
Specialist doctors are available		
Get better treatment		

∗Multiple responses were allowed

### Knowledge of danger-signs and care-seeking for eclampsia

The stories and results of hypothetical ‘case scenarios' on eclampsia are presented below.

Scenario C: Eclampsia during pregnancy

Jasmin was in her ninth month of pregnancy. She had recently moved to her mother's household where she planned for delivering her first baby. Her mother was taking good care of her. However, since the last month, Jasmin experienced periodic severe headaches. Right around her due date, she was not feeling well. One morning, after saying her prayer, she noticed that her vision was blurred. Suddenly, her arms and legs started jerking, and she fell to the ground, convulsing. She bit her tongue between her teeth and made repeated groaning sounds. Once the jerking stopped, she seemed unaware of her surroundings. Her mother inside the household preparing the morning meal heard her fall and ran to her side.

Scenario D: Eclampsia during labour and delivery

During the later stage of her pregnancy, Morzina had developed swelling in her hands and feet, which were painful at times, interfering with her ability to work. She also developed a headache right around her due date. Two days after her due date, she suddenly went into labour. An hour later, her body started convulsing violently and foam came from her mouth. After the seizure, she started groaning.

Table 6 shows the results of hypothetical case scenarios on eclampsia in both the sites. Causes associated with the condition are cold, high blood pressure, malnutrition, and evil spirit. The symptom most likely to inspire action to seek care was ‘convulsions’.

**Table 6.** Perceptions of causes and signs/symptoms of eclampsia by women of reproductive age based on hypothetical case scenarios, Sylhet and Jessore, 2006

Cause, sign/symptom∗	Sylhet (n=20)	Jessore (n=20)
Physical		
Due to cold	8	--
Physical weakness due to malnutrition	5	12
Due to high blood pressure	5	--
Patient did not take tetanus injection during		
pregnancy	--	2
Do not know	--	3
Evil spirit	3	2
Recommended first treatment		
Home	14	10
Facility	6	10
Specific sign and symptom inspire to take step∗		
See seizure/convulsion	11	12
Seizure with biting tongue	4	4
Seizure with foaming from mouth	4	--
Seizure with groaning sound	--	4
Seizure with other physical sign (bending, twisting hand-leg-body)	2	3

∗Multiple responses were allowed

Treatment for eclampsia was first administered in the home, with a village doctor called to the household to administer care if it were perceived to be needed (Table 7). Reasons given for choosing a village doctor were that they lived in close proximity, were available at night, and could start immediate treatment. Other respondents felt that the patient should be transferred to a hospital setting, most specifying a government hospital that offers care by specialized doctors at an affordable price. In both the sites, most respondents indicated that action should be taken within half an hour of the convulsion.

**Table 7.** Perception of care-seeking associated with eclampsia by women of reproductive age based on hypothetical case scenarios, Sylhet and Jessore, 2006

Care-seeking	Sylhet (n=20)	Jessore (n=20)
How quick should action be taken		
Within one hour	2	--
Within half an hour	8	10
Within 5 to 15 minutes	5	6
Instantly	5	4
What type of practitioner chosen and why∗		
Home		
Call an MBBS doctor	3	2
Provides good treatment		
Available		
Call village doctor (not medically trained)	8	7
Available and staying close to home		
Available at night		
Starts immediate treatment		
Call huzur (Muslim spiritual healer who gives spiritual blessings)	2	2
Helped cure from evil spirit		
Stay closer from home		
Call kobiraj (traditional healer, usually provides herbal treatment)	--	1
Available, provides good treatment to cure from		
evil spirit		
Facility		
Go to hospital (Upajila Health Complex/medical college)	8	7
Getting treatment at a lesser cost		
Doctors are available		
Getting specialized doctors		
Go to clinic	--	1
Get specialized doctors		

∗Multiple responses were allowed

### In-depth interviews: recognition of and response to PPH and eclampsia

#### Postpartum haemorrhage

##### Recognition of complication

In both the sites, all the respondents who experienced PPH reported that the prime cause of bleeding was retained placenta or some part of the placenta remaining inside the uterus. Eight of nine women from both the sites had the delivery in the home with a *dai* and the remaining respondent delivering in the home without a birth attendant. In Sylhet (n=05) and Jessore (n=02), women recognized retained placenta as a possible sign of complication, and they became more certain about the complication when bleeding started. Other related signs, such as the woman turning pale and unconscious (n=08) and seizures (n=01), influenced the family to shift the patient to a nearby health facility for treatment. In both the sites, the patient arrived in the facility within 4-26 hours after recognition of the complication. Most respondents reported that their relatives, mothers-in-law, aunts-in-law, and the birth attendant perceived bleeding to be a normal phenomenon that occurs during delivery. Many explained that it is good for a woman to be rid of the blood and fluids because they are associated with pollution.

One birth attendant in Jessore mentioned:

It is normal to bleed after delivery as it helps pour out impure *(kharap)* blood from a mother, which she bore for 10 months and 10 days. Therefore, much bleeding is normal after delivery. It is nothing to worry about, and the amount of bleeding is usually a little more than menstruation.

### Decision to seek care

In such a situation, a mother must rely on influential, elderly family members who usually make decisions relating to childbirth. As bleeding is not considered a serious complication, the decision to take the woman to facility was often delayed. Even when families recognized the complication, patients often could not be transferred to a facility in Sylhet because it was far away; no male family members were present to accompany the woman to the facility; and it was difficult to arrange transportation at night. Similar delays in decision-making took place in Jessore as the respondents and their relatives considered bleeding not to be a serious complication. In addition, the distances to healthcare facilities in Sylhet were greater than in Jessore.

### Actual care-seeking pattern

*Home level:* The respondents in both Sylhet and Jessore first received treatment in the home. A common pattern in both the sites was that when the placenta did not expel normally, the *dai* would enter her bare hand into the uterus to pull the placenta out. In some cases, hair was put into the mother's mouth to induce vomiting, which is believed to induce pressure and help deliver the placenta. However, when these attempts failed, families called a village doctor to their home or purchased medicine from a homeopathic doctor to increase the pain to deliver the placenta. The village doctor (3 cases in Sylhet and 2 cases in Jessore) was often summoned to the home to administer an injection to increase pain so that the placenta comes out. Only in one case in each site, a skilled nurse was called to the home to provide care. Relatives of women who had experienced PPH reported that they thought of transferring the patient to a hospital when treatment in the home proved to be ineffective.

*Facility level:* When treatment in the home was perceived to be ineffective, the patient was shifted to a government or private hospital. After transfer, three of five respondents in Sylhet needed urgent blood transfusion; two of these respondents each needed two bags of blood, and the third needed one bag. These five patients sought care from a government hospital whereas three of four patients in Jessore chose private clinics, which, they indicated, provided better and faster service. Eventually, all the respondents in Jessore were referred to a government hospital as their condition was critical. The respondents reported that these private health service providers preferred not to take responsibility for patients in such a critical condition. At such a crucial stage, such shifting and re-shifting caused harm to a patient as it took valuable time in accessing health services.

In Jessore, one of the four respondents went directly to a government hospital from where she was referred to a higher-level government health facility (medical college hospital). On the way, the driver of the ambulance of the government hospital convinced the family members to transfer the patient to a private clinic, as he explained that it had blood available for transfusions and provided better services. In both the sites, patients were administered saline injection, given tablets to stop bleeding and were transfused blood.

**Fig. 1.**
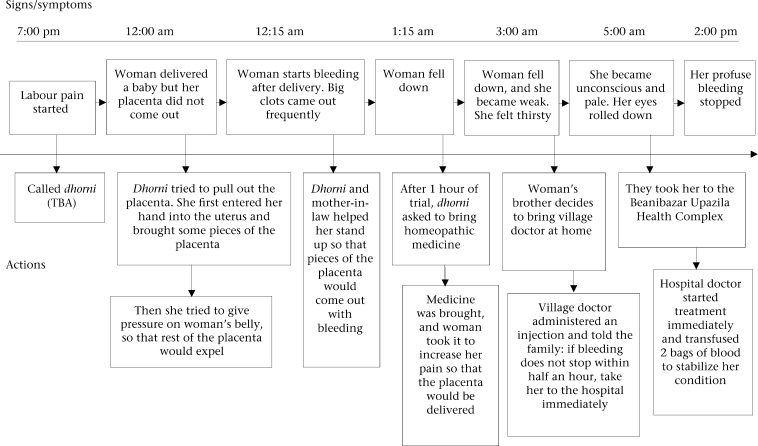
Care-seeking pattern of a woman from Sylhet (low-performing area) with PPH

The case study on the next page (Fig. 1) describes an extreme case, illustrating the obstacles women face in reaching a facility with skilled care. The situation occurred in a remote village of Zakiganj, Sylhet, 55 km from Beanibazar where the nearest comprehensive EOC service provider with facilities for blood transfusions is located. It took two and a half hours for the patient to reach the hospital. In this case, 19 hours passed to stop the bleeding after the labour pain had started. The case study highlights the recognition of the complication, decision-making, and subsequent care-seeking to a higher health facility centre.

PPH=Postpartum haemorrhage; TBA=Traditional birth attendant

### Eclampsia

#### Recognition of complication

In Sylhet, causal explanations associated with eclampsia included evil spirits (four respondents), high blood pressure (three respondents), and blood deficiency (two respondents), and one respondent was uncertain of the cause. In Jessore, three respondents associated the condition with high blood pressure and a swollen body, three said that it was linked to malnutrition, and two respondents attributed the condition to evil spirits. The remaining two were not certain about the cause.

Relatives attending the woman became certain about the severity of the condition when, in addition to convulsing, the woman started foaming at the mouth and bending down on her hands and legs.

One relative of the patient from Jessore remarked:

Everything was going fine … my daughter-in-law told me that she was having headaches after lunch. I told her to lie in bed and take rest. Five minutes later when I came back to the room, I found that she was having convulsions, and her legs and heads became curved. We did not wait much longer and took her to the hospital just after this.

#### Decision to seek care

In Sylhet, patients were transferred one to one and a half hours after the convulsions began. In three cases, the response was delayed; one of these cases reported that convulsions occured due to *dushi* (evil spirits), and therefore, the patient would not be cured if she were transferred to a hospital. They rather felt that the patient needed home-care from a huzur (spiritual healer). Two respondents from Sylhet reported that distance, lack of transportation, and the fact that the condition occurred at night were the reasons for not taking the patient to a health facility earlier. In contrast, all patients in Jessore were transferred to a hospital within 30 minutes after the convulsions started.

#### Actual care-seeking pattern

*Home level:* There were major differences in care-seeking patterns in Sylhet and Jessore for eclampsia. In Sylhet, family members first administered treatment in the home, involving rubbing warm oil on the woman's body just after convulsions started. In two cases, a village doctor was called to administer care after the convulsions started. In another three cases, the woman's family members brought blessed water from a *huzur* as they believed that *dushi* was the reason for the convulsions. Only when these attempts failed, did they think of taking the woman to a hospital.

In Jessore, family members also called in a village doctor or Family Welfare Visitors (FWV) to apply treatment in the home when the patient was experiencing headache, nausea, and hazy vision just before the convulsions began. They shifted the patient to a health facility after the convulsion had started.

*Facility level:* All the five respondents in Sylhet first went directly to the government facility (Upazila Health Complex), and two of them were later referred to a higher government health facility as the women's condition was considered critical.

Data showed that five attendants of the patients in Jessore took the patients to a hospital within half an hour of the onset of convulsions. In these cases, the healthcare provider checked the blood pressure of the patient and subsequently referred the woman to a hospital. Notably, three of the five respondents in Jessore first went to a private health facility and received service there, as they thought that they would not receive quick service at the government facility. Two respondents went directly to a government hospital for treatment but when they found that they were not getting service quickly, they went to a private clinic. Most eclampsia patients stayed in the health facility for 4-5 days. In both the areas, the patients were unconscious for 3-4 days.

**Fig. 2.**
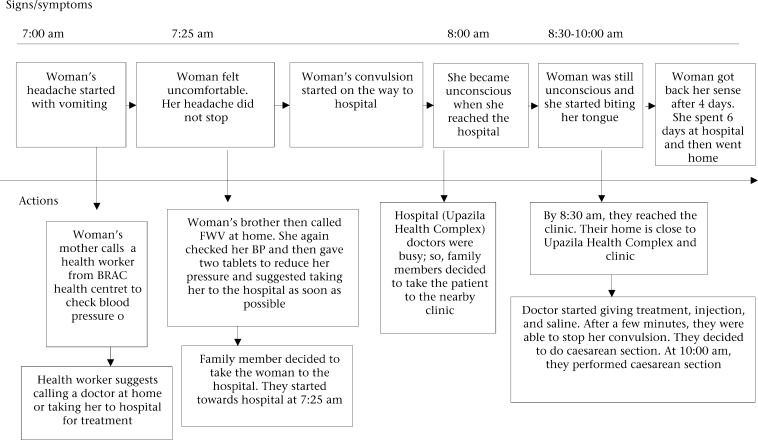
Care-seeking pattern of a woman in Jessore (high-performing area) with eclampsia

A case scenario describing a respondent in Keshobpur, Jessore, is presented in Figure 2. In this case, the Keshobpur government health complex is 30 minutes away by rickshaw from the respondent's home and is, thus, relatively accessible. Private clinics also are available in the area so that people can avail of services from the government or private service providers in the case of complications.

BP=Blood pressure; FWV=Family Welfare Visitor

## DISCUSSION

The results of the study highlight multiple factors that affect care-seeking for two severe maternal complications, including the recognition and perceived severity of the condition and accessibility to health facilities. The findings also illuminate how differences in the sociocultural context may influence the willingness of family members to ensure that appropriate healthcare is availed of when women are in a life-threatening situation.

Women in both low-performing Sylhet district and high-performing Jessore district identified PPH and eclampsia as life-threatening complications. However, there were variations in prioritizing complications by area and age-group of women. Women of reproductive age in the Sylhet region considered PPH a more severe complication than eclampsia. The situation was reversed in Jessore where women from the same age-group perceived eclampsia to be more severe.

Women of reproductive age from both the areas appeared to have a basic understanding of how to treat these complications. The data suggest that they knew when and where to take a woman with complications for better treatment and greater likelihood of survival. In the real-life case studies, however, there were major differences between their understanding of the conditions and their actual care-seeking behaviours in response to both PPH and eclampsia.

In Jessore, there was rapid transfer of women who had suffered eclampsia to a healthcare facility once the condition was recognized as serious. In comparison, patients in Sylhet were not shifted to a health facility quickly as people there perceived eclampsia to be associated with evil spirits. For PPH, there were delays in both the areas in reaching the appropriate level of healthcare facility. While exploring reasons behind the delay, it was found that the respondents and family members considered bleeding as a normal sign of delivery. In addition, in Sylhet, there was a delay in accessing the facility even after the recognition of the complication due to difficulties in locating transport and the distance to reach the health facility.

Results of studies also showed that many women and their elderly female family members did not spontaneously think of PPH as a serious problem (1). Bleeding is viewed as a normal transition that occurs after delivery. The blood remaining in the womb after producing a foetus is thought to be bad blood that needs to be washed out to regain health and a state of purity. Therefore, rather than perceiving bleeding as a problem, women view a lack of bleeding as problematic (16). The respondents in Sylhet and Jessore also indicated that, during the postpartum period, they must expel the impure blood from the womb to regain health.

The sociocultural context in the Sylhet area is quite different from that in Jessore. Women living in Sylhet are less educated than those in the Jessore region; a lower level of education is likely to make women in Sylhet more dependent on decisions of other family members. Moreover, people in the Sylhet region are known to be more conservative, which influences women's movement outside the home to be highly restricted. Women in Sylhet are less willing to go to health facilities often out of fear that male service providers might attend them. In addition, service providers in the highly-conservative Sylhet region are mostly non-local, posing extra social distance between care providers and patients and their family members. Adding to these constraints, the distance between homes and the EOC facility is greater in Sylhet compared to Jessore.

In Jessore, the same barriers to accessing care were less significant or absent. For instance, the organization of EOC services, including the distribution and functionality of facilities and availability of trained care providers is better in the Jessore region (1). In addition, higher education of women and a less-conservative outlook appeared to allow women greater mobility and accessibility to healthcare facilities. In summary, both sociocultural context and availability of healthcare appeared to influence the higher performance in the Jessore region.

In the main results, however, no notable difference was found in the general care-seeking patterns in both the sites. Rather, when responding to both the types of complications, a common phenomenon is first to apply treatment in the home. Typically, family members of a patient rely on and seek care from village doctors, herbalists, religious healers, and practitioners who have been living in the locality for a long time and with whom they have a relationship.

The challenge now is to overcome some major social and physical barriers, e.g. distance, transportation, restricted movement of women, etc., women face in accessing delivery care. An important next step is to create greater social awareness among respondents, e.g. elderly family members and husbands, through interventions in low-performing areas and motivating families to avail of healthcare in time from skilled health attendants and from comprehensive EOC facilities during complications. In a low-performing area, more EOC facilities are needed allowing these to be more accessible to families. More research is also needed on recognition of bleeding as a normal phenomenon and how the seriousness of excessive bleeding can be communicated to families. Future investigations also need to look into reasons for greater acceptability of private service providers compared to government service providers in Jessore.
